# Low-Temperature Plasma Modification of Styrene–Butadiene Block Copolymer Surfaces for Improved Adhesion—A Kinetic Approach

**DOI:** 10.3390/polym12040935

**Published:** 2020-04-17

**Authors:** Jacek Tyczkowski, Hanna Kierzkowska-Pawlak, Jan Sielski, Iwona Krawczyk-Kłys

**Affiliations:** 1 Department of Molecular Engineering, Faculty of Process and Environmental Engineering, Lodz University of Technology, Wolczanska 213, 90-924 Lodz, Poland; hanna.kierzkowska-pawlak@p.lodz.pl (H.K.-P.); jan.sielski@p.lodz.pl (J.S.); 2 Institute of Leather Industry, Zgierska 73, 91-462 Lodz, Poland; iwonka.krawczyk@gmail.com

**Keywords:** SBS copolymer, plasma surface modification, kinetics, adhesion, oxygen functional groups, roughness, peel test

## Abstract

This paper proposed a kinetic model that can describe the changes in the adhesion properties of styrene–butadiene (SBS) block copolymer surfaces under the influence of low-temperature plasma treatment. As a measure of these changes, the peel strength of joints formed between the copolymer surface and the polyurethane adhesive was chosen. Five types of low-temperature low-pressure RF plasma, two inert plasmas (Ar and He), and three reactive plasmas (O_2_, CO_2_, and CCl_4_) were tested. It was found that for all these types of plasma, the peel strength with the plasma treatment time first increases rapidly reaching a maximum value, and then there is a visible decrease in peel strength, after which the peel strength increases again. This dependence of the peel strength on the plasma treatment time is very well described by the proposed model, which considers three processes: (1) the generation of radical states followed by the creation of functional groups involved in the adhesive bonding process, (2) the surface cross-linking that decreases the concentration of these functional groups, and (3) the formation of nano-roughness. The model analysis revealed differences between the action of reactive and inert plasmas in the SBS surface cross-linking mechanism and preferential etching process, as well as differences in the generation of radical states between the O_2_ plasma (electron process) and other plasmas tested (ionic processes).

## 1. Introduction

In general, the adhesive bonding of polymers to themselves as well as to other materials presents an important problem, especially in the case of elastomers and rubbers used in the industry. The strength and quality of such adhesive-bonded joints depend, to a great extent, on the chemical structure and morphology of the polymer surface, and therefore can be controlled by various surface treatments. It is already known for more than 20 years that a particularly useful method in this respect is the low-temperature (non-equilibrium), both low pressure and atmospheric pressure, plasma treatment [1,2,3,4,5,6,7]. Such treatment, which can be performed either in inert plasmas (generated e.g., in Ar or He) or chemically reactive (but non-polymerizable) plasmas (generated e.g., in O_2_, CO_2_, or H_2_O), causes changes in the chemical structure of the polymer surface by two fundamental processes. The first one consists in the cleavage of chemical bonds on the surface, which leads to the creation of radical states. On the states, functional groups, such as hydroxyl (−OH), carbonyl (>C=O), etc., can be formed directly in the plasma processes (from atoms present in the reactive plasmas) or further after the contact of the surface with air atmosphere (in the case of inert plasmas). The radical centers are also responsible for the formation of cross-linking bonds (for example, C−C, C−O−C), the number of which increases with the concentration of these centers. In turn, the second process involves bombarding the surface with ions, which leads to the preferential etching of the surface by removing certain atoms or their groups and, consequently, increases the surface roughness [4].

Usually, after the plasma treatment, a pronounced increase in the adhesion of the polymer surfaces to adhesives was observed. In some cases, only a few seconds of plasma exposure was enough to obtain several times higher peel strength than that of the non-treated samples [4]. There is no doubt that the observed improvement in adhesive properties results from changes in the chemical structure and nanomorphology of the polymer surface. It was found that the functional groups produced on the surface, on the one hand, can react chemically with adhesives, causing the chemical adhesion [8], and on the other hand, they can enhance the surface energy (mainly the polar component of the surface energy) being responsible for the thermodynamic adhesion [9]. The surface roughness generated during plasma treatment leads in turn to the enhancement of the mechanical adhesion [10,11]. Thus, it would be expected that the adhesion force should steadily increase with increasing plasma treatment time. However, it has already been reported several times that after some time of plasma treatment, the peel strength of the adhesive-bonded joints formed with the polymers begins to decrease during further processing [9,12,13,14,15]. Besides, it was also observed that for longer treatment time, after the reduction of the peel strength, its re-increase occurred [13].

The aim of this work is an attempt to explain, based on the kinetic approach, changes occurring on the polymer surface during the plasma treatment. As a measure of these changes on the surface, the peel strength of the adhesive-bonded joints was established. It was assumed that the value of this parameter is directly proportional to the concentration of active states responsible for the adhesion, such as appropriate functional groups and roughness. The studies were carried out on a model elastomer such as a styrene–butadiene–styrene (SBS) copolymer. Rubbers based on SBS copolymers are one of the main materials used in the footwear industry for the production of soles. Therefore, there is a wide interest in gluing these materials (mainly with the use of polyurethane adhesives) [16,17]. Plasma surface activation of elements made of SBS rubbers, which is a clean, waste-free, and environmentally friendly method, is currently the leading solution in this area. However, it requires further research to better understand the mechanisms of plasma activation of the surface, which has a direct impact on the practical application of this method.

## 2. Materials and Methods

### 2.1. Materials

A radial type styrene–butadiene block copolymer Finaprene 435 (F435) (Atofina S.A., Feluy, Belgium), as a model of polymeric material, was selected to investigate the plasma treatment processes. The copolymer was unvulcanized and did not contain any ingredients such as oils, plasticizers, and fillers. The weight ratio of butadiene to styrene was 69 to 31. Samples for peel tests were obtained by an injection of the copolymer into a heated mold at 150 °C, where plates of 120 mm width, 150 mm length, and 6 mm thickness were formed. Then, strips of 15 mm width and 75 mm length were cut from the plates. Samples for FTIR and Atomic Force Microscopy (AFM) measurements were prepared in the same manner, only with a thickness of approximately 0.5–1 mm. All samples were cleaned in an ultrasonic washer (with methanol (pure p.a., POCh S.A., Gliwice, Poland)) for 15 min.

### 2.2. Plasma Treatment

The plasma treatment was performed in a parallel plate reactor with a radio frequency (RF, 13.56 MHz) glow discharge. A detailed description of the reactor is given in [18]. The plasma was generated in a reactor chamber fed by various gaseous compounds that were in the flow. Two types of plasmas were tested—the reactive (O_2_, CO_2_, and CCl_4_) and inert (Ar and He) plasmas. For the preparation of the reactive plasmas, we used gases such as oxygen (pure O_2_, 99.95%, Linde Gaz Polska Ldt., Cracow, Poland) and carbon dioxide (CO_2_, 99.95%, Linde Gaz Polska Ldt., Cracow, Poland), both with a flow rate of 7.5 sccm (standard cubic centimeters) and an initial pressure of 13 Pa, as well as tetrachloromethane vapor (CCl_4_, pure p.a., POCh S.A., Gliwice, Poland) with a flow rate of 3.5 sccm and an initial pressure of 13 Pa. The inert plasmas were generated in argon (Ar, 99.999, Linde Gaz Polska Ldt., Cracow, Poland) and helium (He, 99.999, Linde Gaz Polska Ldt., Cracow, Poland), both with a flow rate of 7.5 sccm and an initial pressure of 13 Pa. The power of the glow discharge was varied from 10 to 80 W, and the plasma treatment of the samples lasted from 5 s to 20 min.

### 2.3. Peel Tests

To determine the adhesive strength of the SBS copolymer surface, 180°-peel tests, according to the European Standard EN 1392:2007, were carried out. Adhesive bonded joints were prepared using the copolymer samples before and after the plasma treatment and strips of standard leather (boxcow, chrome-tanned, non-pigmented). Polyurethane (PU) adhesive (18 wt. % polyurethane pellets (Pearlstick 45-60/14, Merquinsa, Montmeló, Spain) in acetone/toluene, 3:1 (pure, POCh S.A., Gliwice, Poland) + 1.3 wt. % 4,4-methylenediphenyl diisocyanate, (MDI, pure, Huntsman, Osnabrück, Germany)) was spread on each adherend and dried at room temperature for 15 min. The dry adhesive films were activated by heating at 353 K for 3 min, and the surfaces were immediately contacted under a pressure of 0.4 MPa for 10 s. The adhesive joints were then conditioned for 48 h at room conditions. The peel strength measurements were performed using a tensile tester model 5566 (Instron, High Wycombe, UK) at a peel rate = 1.67 × 10^−3^ m/s. The average value of the peel strength for a given type of surface was determined from at least three samples and a minimum of 10 measurement points for each of them.

### 2.4. FTIR Spectroscopy

Fourier Transform Infrared–Attenuated Total Reflectance Spectroscopy (FTIR-ATR) spectra were recorded in the 4000 to 400 cm^−1^ range using a Jasco FT/IR 6200 spectrometer (JASCO Inter. Co., Ltd., Tokyo, Japan) equipped with an MCT M detector cooled by liquid nitrogen (77 K) and a MIRacle ATR sampling accessory (diamond/ZnSe) (PIKE Technol., Madison, WI, USA). The whole spectrometric system was purged by dry argon. The scanning rate was 0.1 cm/s. Signal accumulation from 300 scans was taken with a resolution of 1 cm^−1^. Interesting parts of the spectra were analyzed according to a numerical peak-fitting algorithm (PeakFit™, Systat Software Inc., San Jose, CA, USA).

### 2.5. Atomic Force Microscope (AFM)

For the topographical analysis of the SBS surfaces with and without plasma treatment, an atomic force microscope (Nanoscope 3D, Veeco Instruments, Santa Barbara, CA, USA) was used. The surface observations were made in the tapping mode. Images were taken for areas of 1–20 μm in a two-dimension (2D) scale. The measurements were performed using a standard RTESP-type silicon probe (Bruker AFM Probes, Camarillo, CA, USA). The average surface roughness (in nanometers) was estimated by the Roughness routine of the Nanoscope Software 6.13 (Veeco Instruments), using the R_a_ parameter, which is the arithmetic average of the absolute values of the surface height deviations measured from the mean plane. The 16.8 μm × 16.8 μm area was scanned with a scan rate of 0.15 Hz.

## 3. Results and Discussion

**Figure 1:**
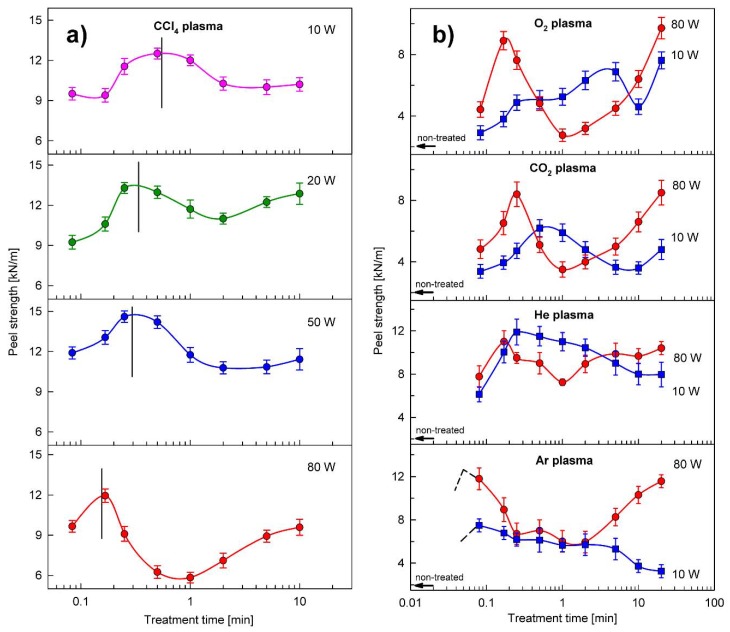
Peel strength measurements for the styrene–butadiene–styrene (SBS) copolymer treated by various types of plasma as a function of the treatment time and the discharge power (the lines are for visual guidance): (**a**) CCl_4_ plasma; (**b**) O_2_, CO_2_, He, and Ar plasmas. Treatment time is presented on a logarithmic scale to more accurately show the changes over the entire treatment period. The peel strength for the non-treated SBS surface is 2.0 ± 0.2 kN/m.

For a more profound understanding of the polymer surface modification created by low-temperature plasma treatments, we analyzed the dependences of the peel strength for adhesive-bonded joints prepared between the SBS surface and PU adhesive on the plasma treatment time and discharge power using various types of plasma. Figure 1a presents an example of such dependences for the CCl_4_ plasma. Similar dependences for other types of plasma, but only for selected discharge powers (extreme values), are shown in Figure 1b. As can be seen, all plasma treatments, irrespective of the type of plasma and the discharge power, produce a considerable improvement in the adhesion properties of the SBS copolymer. The peel strength increases rapidly and attains a maximum value in a relatively short time of plasma treatment. (Although this is not seen in the Ar plasma (Figure 1b), there is no doubt that also in this case the maximum exists somewhere in time ≤ 5 s, because for time = 0 (without plasma treatment) the peel strength is only ~2.0 kN/m.) Then, a decrease in the peel strength value is visible, after which in most cases, especially for high power discharges, the peel strength increases again. Although all these relationships have the same character, their exact shape depends on the type of plasma and the discharge power. For example, the maximum value of the peel strength occurring in the initial plasma treatment period clearly shifts towards shorter times with an increase in the discharge power, but in different ways for each type of plasma used.

To explain the nature of the observed phenomena, it should first of all be emphasized that as the peel strength depends on the parameters of the plasma treatment, it proves that the SBS–PU interface is responsible for the peel test results. If SBS, PU, and leather cohesion forces or the PU–leather interface were responsible for the peel strength, peel strength should be independent of the plasma treatment and should be practically constant.

**Figure 2:**
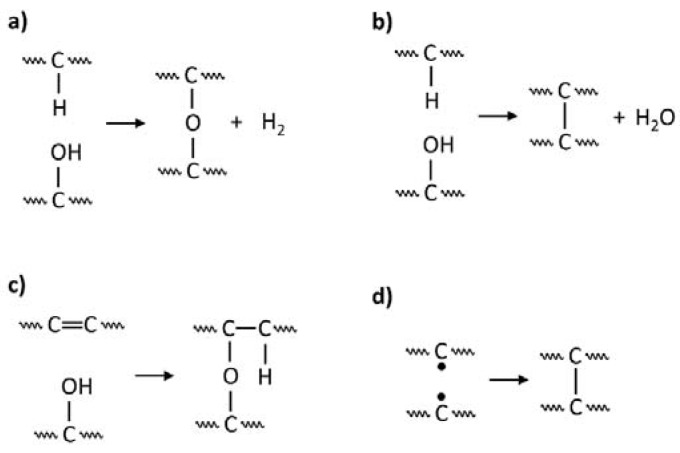
An example of plasma-induced cross-linking processes (**a**–**c**) involving C−OH groups and (**d**) direct cross-linking between the radical states. Proposed on the basis of, inter alia, the works in [19,20,21].

For the description of the peel strength changes as a function of the plasma treatment time, three main processes were considered: (1) the generation of radical states followed by the creation of functional groups involved in the adhesive bonding process, (2) the surface cross-linking that decreases the concentration of these functional groups, and (3) the formation of nano-roughness. In our case, the first process concerns the generation of radical states on the surface and then creating functional groups on them, such as C−OH, C−Cl, and >C=O, capable of reacting with the PU adhesive. As already mentioned, in reactive plasmas, these groups are formed directly during the plasma treatment, whereas in inert plasmas, they are formed mainly after contacting the radical states with air. The second process involves the reduction of both the existing functional groups and the sites where they could be created. In reactive plasmas, this consists in converting in plasma the already formed functional groups into groups that are inactive in relation to the adhesive (mainly as a result of crosslinking). An example of such predicted reactions for C−OH groups is shown in Figure 2a–c. In turn, the reduction of the sites where functional groups can be created is effected by direct recombination and cross-linking between the radical states. This effect dominates in the case of inert plasmas before the polymer surface is contacted with air and the formation of functional groups begins (Figure 2d). Thus, regardless of the type of plasma, we can observe in the second process a systematic decrease in the concentration of active functional groups that can react with the PU adhesive. The higher the concentration of radical states and/or functional groups, the easier the cross-linking process with their participation occurs.

The course of the first and second processes justifies the assumption that they are consecutive processes, each of the first order. For constant plasma parameters, the rate of the first process depends only on the concentration of surface sites ready to form radical states (for example, ~C−H → C^•^). The rate of the second process, in turn, depends only on the concentration of created radical states. The resulting balanced concentration of radical states after a given plasma treatment time is, in fact, equal to the concentration of active functional groups formed on these states (during the plasma process or after contact with air), which in turn is proportional to the peel strength (*F_p_*’) based on the interaction between the functional groups and the PU adhesive. The integrated form of the kinetic equation describing these two consecutive processes (by analogy to the commonly known equation for the consecutive reactions, see, e.g., in [22]) is as follows,

(1) $$F_{p}^{’}=A_{0}\frac{k_{1}}{k_{2}-k_{1}}\;\left(e^{-k_{1}t}-e^{-k_{2}t}\right),$$

where *k*_1_ and *k*_2_ are the rate constants for the first and second processes, respectively; *A*_0_ is a parameter proportional to the initial concentration of surface sites, on which radical states can be created; and *t* is the plasma treatment time.

**Figure 3:**
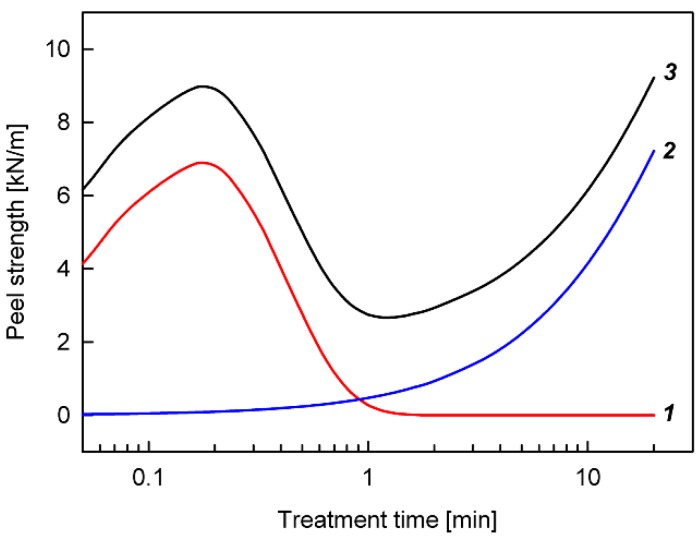
Curves showing the theoretical relationships between the peel strength and the plasma treatment time in the proposed kinetic model, for example, with assumed values of parameters similar to those determined experimentally (see Table 1): (curve 1) according to Equation (1) with *A*_0_ = 18.5 kN/m, *k*_1_ = 6.1 1/min, and *k*_2_ = 5.95 1/min; (curve 2) according to Equation (2) with *B*_0_ = 16 kN/m and *k*_3_ = 0.03 1/min; (curve 3) according to Equation (3) with *F*_0_ = 2 kN/m. (Curves 2 and 3 will be discussed later in the text.)

An example of the theoretical curve described by Equation (1) is shown in Figure 3 (curve 1). As one can see, there is a clear maximum resulting from the competition between the process leading to the formation of functional groups and the process leading to their reduction. Such maxima, as already shown above, are observed for the dependence of the peel strength for the adhesive-bonded joints on the plasma treatment time.

**Figure 4:**
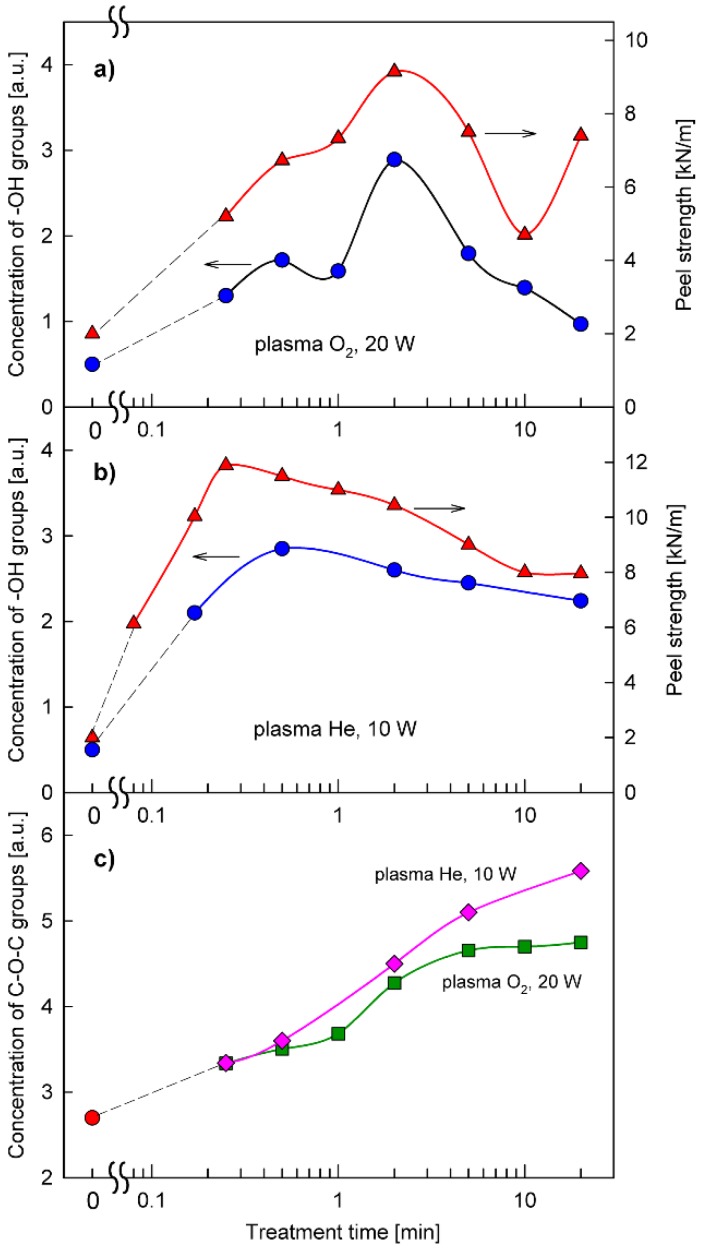
Peel strength and SBS surface concentration of −OH and C−O−C groups as functions of plasma treatment time: (**a**) correlation between peel strength (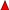) and −OH concentration (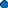) for O_2_ (20 W) plasma; (**b**) correlation between peel strength (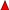) and −OH concentration (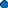) for He (10 W) plasma; (**c**) the dependence of C−O−C concentration on the time of treatment with O_2_ (20 W) plasma and He (10 W) plasma.

Indeed, it has already been found several times that changes in the concentration of functional groups responsible for reactions with PU adhesives (C−OH, C−Cl, >C=O), measured directly by FTIR and XPS spectroscopies as a function of plasma treatment time, demonstrate a good correlation with an analogous dependence for the peel strength of adhesive joints [4,8,13,18]. Figure 4a,b shows examples of the relationships of the −OH groups’ concentration and the peel strength as functions of treatment time with O_2_ plasma and He plasma, respectively.

**Figure 5:**
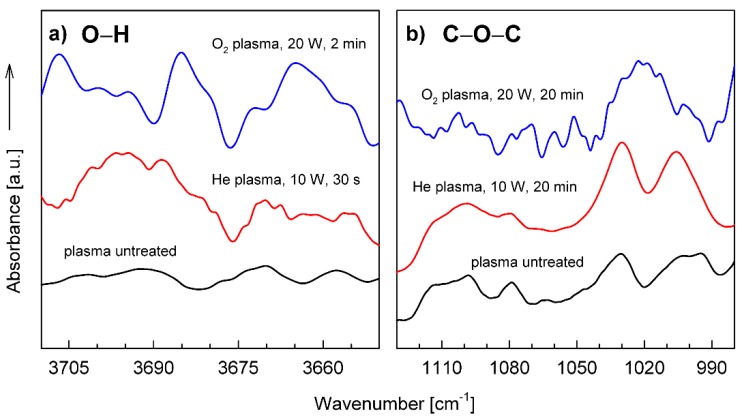
Fourier Transform Infrared–Attenuated Total Reflectance Spectroscopy (FTIR-ATR) spectra for the SBS copolymer untreated as well as O_2_ and He plasmas treated, presented in the regions characteristic of O−H and O−C−O vibration bands: (**a**) the plasma treatment times correspond to obtaining the maximum peel strength for a given plasma; (**b**) the spectra are shown for the longest plasma treatment time used.

The relative concentrations of −OH groups are expressed by normalized surface areas of the characteristic IR bands in the range of 3650 to 3710 cm^−1^, which are assigned to O−H stretching vibrations [23]. This range of IR spectrum is shown in Figure 5a. As can be seen, the positions of the maxima on the plasma treatment time scale for the concentration of −OH groups and the peel strength of adhesive joints are in good agreement with each other. On the other hand, it was also found that the relative concentration of C−O−C bridges predicted as one of the results of the cross-linking process (shown in Figure 2), revealed by IR bands in the range of 990 to 1130 cm^−1^ (Figure 5b), which are attributed to C−O−C stretching vibrations [23], increases systematically with the plasma treatment time. Thus, the presented results of the IR investigations are fully consistent with the concept of two consecutive processes described by the kinetic Equation (1): the process of creation of functional groups and the process of their reduction.

**Figure 6:**
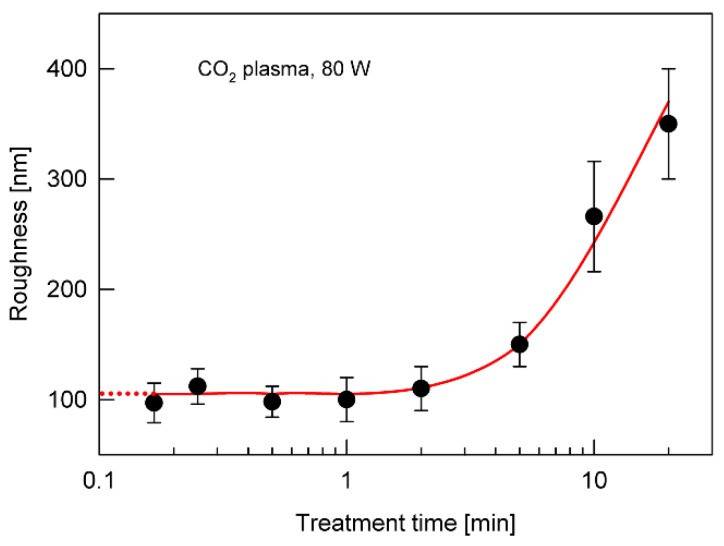
Changes in the surface roughness of the SBS copolymer treated with CO_2_ (80 W) plasma at different times (based on Atomic Force Microscopy (AFM) measurements).

**Figure 7:**
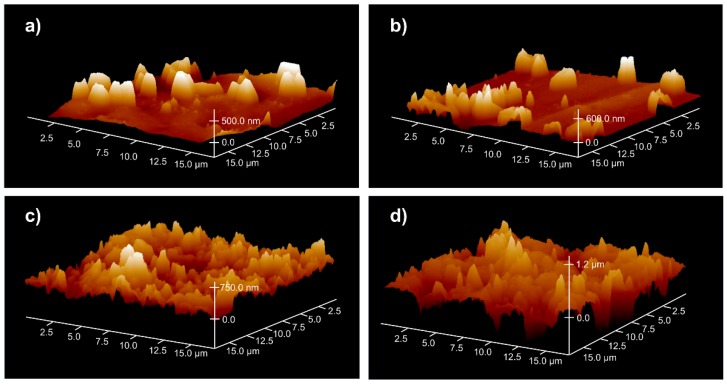
AFM images illustrating changes in roughness on the SBS surface treated with CO_2_ (80 W) plasma at different times: (**a**) non-treated; (**b**) 1 min; (**c**) 5 min; (**d**) 20 min.

The third process contributing to the strength of the adhesive joint (*F_p_*’’) is the process of creating roughness on the surface as a result of preferential etching. For fixed plasma parameters, that is, for a fixed reaction rate constant, the rate of this process depends only on the concentration of the sites on the surface susceptible to the etching, and therefore the process can be described by the following first-order equation,

(2) $$F_{p}^{\prime \prime }=B_{0}\;\left(1-e^{-k_{3}t}\right),$$

where *k*_3_ is the rate constant of the third process and *B*_0_ is a parameter proportional to the initial concentration of sites on the surface, which are involved in the formation of roughness. An example of the theoretical curve described by Equation (2) is shown in Figure 3 (curve *2*). The experimental evaluation of surface roughness as a function of the plasma treatment time confirms the nature of this relation. Figure 6 presents an example of the roughness changes on the SBS surface subjected to plasma treatment, determined from AFM measurements. In Figure 7, several selected AFM images illustrating such changes in the roughness are shown.

Taking into account all three processes discussed above, the dependence of the total peel strength of the adhesive-bonded join (*F_p_*) on the plasma treatment time can be described by the overall kinetic equation, which is a combination of Equations (1) and (2). For formalities, the peel strength for the plasma non-treated surface (*F*_0_) is also included in the equation

(3) $$F_{p}=F_{0}+F_{p}^{\prime }+F_{p}^{\prime \prime }=F_{0}+A_{0}\frac{k_{1}}{k_{2}-k_{1}}\;\left(e^{-k_{1}t}-e^{-k_{2}t}\right)+B_{0}\;\left(1-e^{-k_{3}t}\right).$$

The theoretical curve described by Equation (3), which is a combination of curves 1 and 2, is shown in Figure 3 (curve 3). As one can see, its shape is very similar to the experimental relationships between the peel strength and the plasma treatment time (Figure 1). Equation (3) was therefore used to analyze all experimental results concerning the dependence of *F_p_* on *t* obtained for the O_2_, CO_2_, CCl_4_, Ar, and He plasmas. It was done according to a numerical curve-fitting algorithm (TableCurve, Jandel Sci., USA), which enables to determine the parameters *k*_1_, *k*_2_, *k*_3_, *A*_0_, and *B*_0_. In general, very good fitting between the experimental results and the theoretical curves calculated from Equation (3) was obtained. The determined parameters and the coefficient of determination *r*^2^ are given in Table 1.

Several important conclusions can be drawn from the data presented in Table 1. First of all, the *k*_1_ values are drastically higher for the Ar plasma than those for all other plasmas used. This indicates that the Ar plasma, in contrast to the other plasmas, is much more effective in generating free radicals on the SBS surface. On the other hand, the rate constant for the cross-linking process (*k*_2_) behaves differently depending on the type of plasma. For the reactive plasmas (generated in O_2_, CO_2_, and CCl_4_), the *k*_2_ values are very similar to the corresponding *k*_1_ values. As the rate constants *k*_1_ and *k*_2_ contain information about the mechanism of plasma action, it can be assumed that both processes—the formation of active functional groups as well as their reduction—proceed by the same plasma mechanism. However, for both inert plasmas (generated in Ar and He), the rate constant *k*_2_ has similar values, and they are practically the same for various discharge powers. The lack of dependence between the cross-linking process and plasma parameters could have been predicted taking into account the fact that in this case the direct cross-linking between the created radical states takes place, for which the plasma is no longer needed.

Similarly, the differences between the reactive and inert plasmas are visible in the preferential etching processes. For the reactive plasmas, there is a systematic increase in reactivity (*k*_3_ increases) with increasing discharge power, with the O_2_ plasma being the least reactive. The concentration of sites on the surface (parameter *B*_0_), which are involved in the O_2_ plasma etching process, is almost one order of magnitude greater than when the CO_2_ and CCl_4_ plasma is used, which proves that another etching mechanism is active in the presence of the O_2_ plasma. In the case of the inert plasmas, however, the correlation between the discharge power and the etching process is not as clear and requires further investigations, although the values of *B*_0_ are comparable to the ones obtained for the CO_2_ and CCl_4_ plasma, which may indicate the similarity of etching processes in the plasma of Ar, He, CO_2_, and CCl_4_.

Finally, let us pay attention to the *A*_0_ parameter. Its values, as shown in Table 1, which should characterize the chemical structure of the SBS surface before plasma treatment, are indeed practically independent of the plasma types and discharge powers (*A*_0_ = 14 ± 1 kN/m). This confirms the correctness of the proposed kinetic model. Slightly higher values of *A*_0_ than those of *B*_0_, except for O_2_ plasma, where we anticipate another etching mechanism, confirm the assumption that the concentration of bonds that can participate in the creation of free radicals should be higher than the concentration of bonds involved in the etching process. For example, cleavage of the C−H bond generates a radical center on the surface (C^•^), but it is not yet sufficient to start the polymer chain scission, which initiates the formation of nano-roughness.

**Figure 8:**
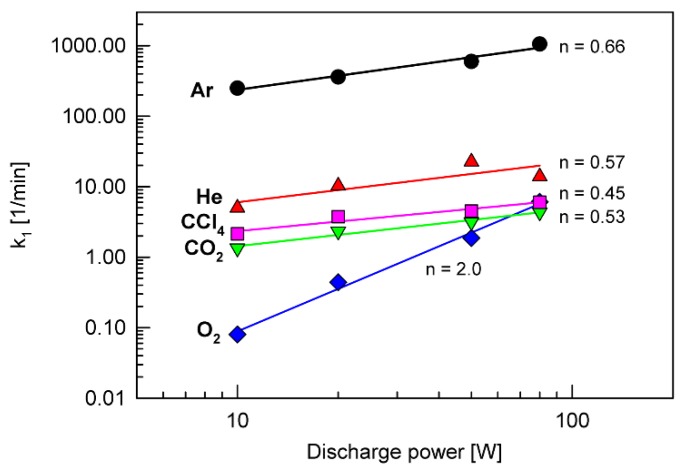
Dependence of the rate constant *k*_1_ on the discharge power for all types of plasma tested, plotted according to Equation (4). The values of the parameter *n* were calculated based on the linear regression of the plots.

To more accurately determine the mechanism of the free radical generation process, the dependence of the rate constant *k*_1_ on the discharge power was analyzed. As a basis, the power function in the following form was taken,

(4) $$k_{1}=a\;P^{n},$$

where *P* is the discharge power, and *a* and *n* are the coefficients of the equation. Figure 8 shows graphs of this dependence for all tested plasma types. In all cases, except for the O_2_ plasma, the coefficient *n* is equal to approximately 0.5−0.6. For the O_2_ plasma, this coefficient reaches a value of 2.0.

Generally, two mechanisms can be responsible for the radical generation on the SBS surface by low-temperature (RF) plasma. The first is connected with the electron bombardment, and the second with the ion bombardment. It has been already found that the dependence of the radical formation rate on the discharge power is approximated by a square function (*n* ≈ 2) when the first mechanism dominates [24]. On the other hand, if the second mechanism determines the radical formation, the rate of the process is proportional to the average energy of ions bombarding the surface, which is, in turn, approximated by a square root function of the discharge power (*n* ≈ 0.5) [25,26]. On this basis, it can be concluded that the ion bombardment mechanism dominates during the SBS surface treatment by inert gas plasmas (Ar and He) as well as CO_2_ and CCl_4_ plasmas. In the case of O_2_ plasma, its interaction with the SBS surface should be associated with the electron bombardment mechanism. The different behavior of the O_2_ plasma compared to other plasma types tested in this work has already been revealed in the SBS surface etching discussed above.

## 4. Conclusions

The studies performed within the scope of the present work bring us closer to understanding the plasma processes taking place on the SBS surfaces. It has been found that three main processes are connected with the plasma treatment: the creation of free radicals on the SBS surface, on which in turn active functional groups are formed; the cross-linking that causes reduction of the functional groups; and the plasma etching enhancing the surface roughness. On this basis, a kinetic model describing the dependence of the peel strength of SBS–PU joints on the plasma treatment time has been proposed. Generally, the model fits the experimental results very well. A more detailed analysis of the model allowed showing of the differences between the action of reactive and inert plasmas, which mainly consist in a different mechanism of the SBS surface cross-linking after the generation of radical states and another mechanism of the preferential etching process. There are also significant differences in the mechanism of generating radical states on the surface where, in the case of the O_2_ plasma, it is associated with the electron process, whereas when treated with other types of plasma (CO_2_, CCl_4_, Ar, and He), the ion process is involved. It is worth noting that among all plasmas tested, the Ar plasma is the most effective in the radical generation process.

The conducted research shows that the maximum value of the peel strength of SBS−PU joints and the time of plasma treatment at which it is achieved is the result of competition between the formation and reduction of active functional groups on the SBS surface. This competition is significantly dependent on the type and parameters of the plasma used. In many cases, plasma treatment time of less than one minute is enough to reach the maximum. This confirms the assumption that the process of creating roughness has a very small impact on the value and position of the peel strength maximum. However, the roughness contribution in the adhesive bonding process is visible in the case of much longer plasma treatment times.

The results obtained in this work are important not only at the basic level, but also for the application, for example, in the plasma bonding technology in the footwear industry. For such industrial applications, one of the most important conclusions drawn from the research presented in this work is the finding that for a given polymer material treated with plasma, there is a specific maximum strength of the adhesive joint, which can be achieved only by careful selection of appropriate parameters of the plasma treatment process. First of all, the type of plasma gas, discharge power, and treatment time must interact together here to ensure the best adhesive bonding result.

## Figures and Tables

**Table 1:** Fitting parameters (according to Equation (3)) for the experimental results concerning the dependence of *F_p_* on *t* obtained for the SBS surface treated with O_2_, CO_2_, CCl_4_, Ar, and He plasmas.

Power (W)	*r* ^2^	*k*_1_ (1/min)	*k*_2_ (1/min)	*k*_3_ (1/min)	*A*_0_ (kN/m)	*B*_0_ (kN/m)
Reactive Plasmas						
**O_2_ Plasma**						
**10**	0.910	0.08	0.10	0.002	13.0	46.0
**20**	0.990	0.44	0.45	0.004	18.8	45.0
**50**	0.998	1.87	1.40	0.011	14.5	30.0
**80**	0.940	6.10	5.95	0.030	18.5	16.5
**CO_2_ Plasma**						
**10**	0.94	1.34	0.60	0.103	11.1	3.07
**20**	0.89	2.33	2.30	0.103	11.4	14.8
**50**	0.93	3.15	3.20	0.16	12.0	5.26
**80**	0.93	4.35	4.20	0.22	13.6	7.15
**CCl_4_ Plasma**						
**10**	0.97	2.14	2.14	0.34	25.5	11.8
**20**	0.96	3.75	3.83	0.83	14.9	10.6
**50**	0.97	4.50	4.41	0.90	9.7	8.94
**80**	0.97	6.00	5.94	1.14	23.0	7.42
**Inert Plasmas**						
**Ar plasma**						
**10**	0.80	250	1.4	2.0	7.2	4.1
**20**	0.93	360	1.6	1.6	10.0	6.5
**50**	0.88	597	4.7	0.84	15.8	8.8
**80**	0.98	1056	1.9	0.35	13.4	11.0
**He Plasma**						
**10**	0.97	5.01	1.0	1.34	15.9	8.3
**20**	0.90	10.3	1.1	0.98	13.2	8.8
**50**	0.98	22.6	0.93	0.25	8.8	10.7
**80**	0.93	13.9	2.2	0.85	11.5	10.1
